# Editorial: Advancing inflammatory bowel disease treatment through nutritional interventions

**DOI:** 10.3389/fnut.2025.1712603

**Published:** 2025-10-20

**Authors:** Nallely Bueno-Hernández, Nalleli Vivanco Karlsson, Viridiana M. Mendoza-Martínez, Aurora E. Serralde-Zúñiga

**Affiliations:** ^1^Proteomics and Metabolomics Laboratory, Research Division, Hospital General de México Dr. Eduardo Liceaga, Secretaría de Salud, Mexico City, Mexico; ^2^Sahlgrenska University Hospital, Gothenburg, Sweden; ^3^Department of Pediatrics, Skaraborg Hospital, Skövde, Sweden; ^4^Clinical Nutrition Service, Instituto Nacional de Ciencias Médicas y Nutrición Salvador Zubirán, Mexico City, Mexico

**Keywords:** inflammatory bowel diseases, Crohn's disease, ulcerative colitis, diet, nutrition

**Figure 1:**
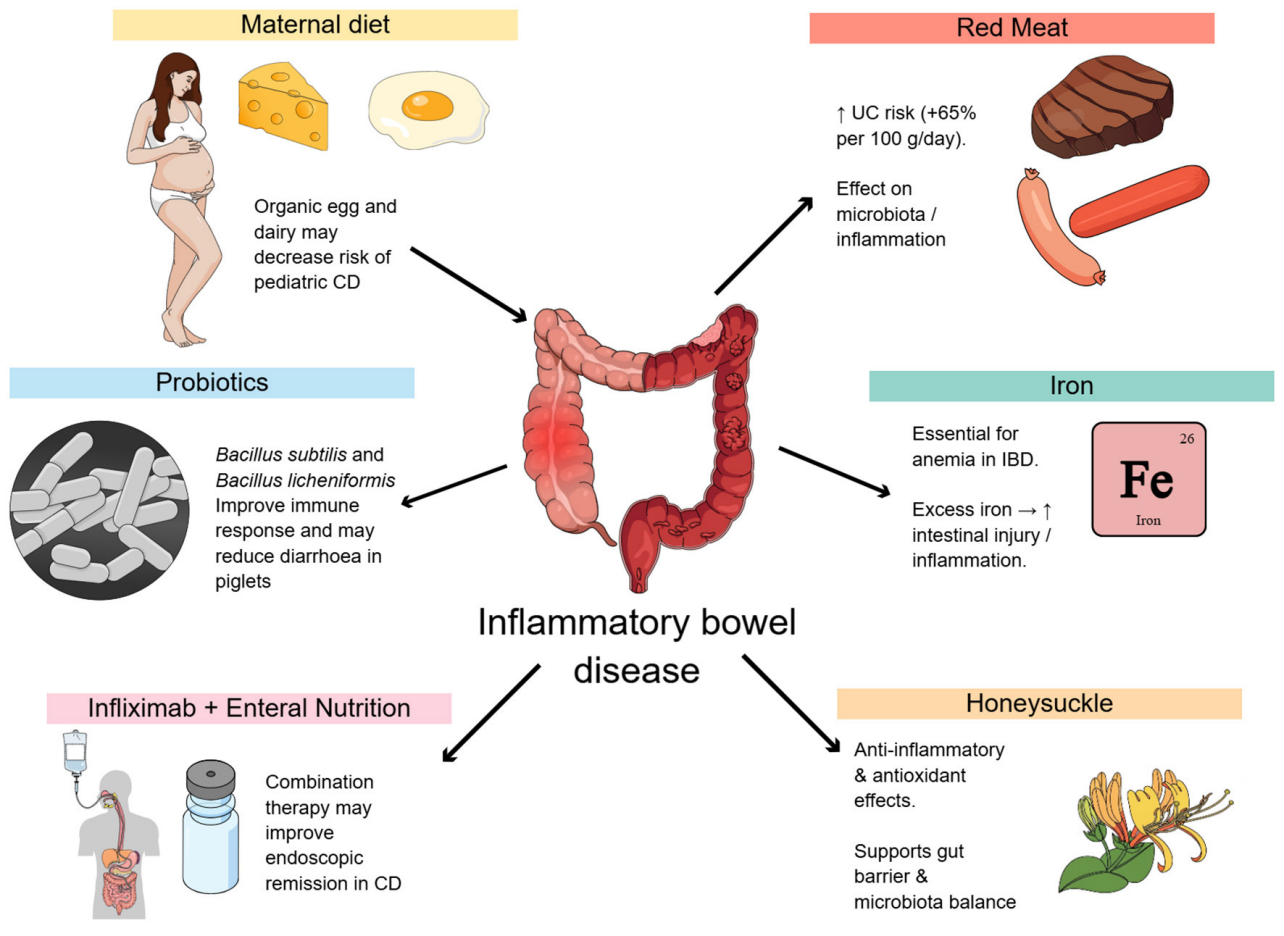
Graphical representation of the effects. IBD, Inflammatory Bowel Disease; CD, Crohn's disease; UC, ulcerative colitis.

Despite significant advances in genetics, Inflammatory Bowel Disease (IBD), including Crohn's disease (CD) and ulcerative colitis (UC), is still a complex and multigenetic disease whose incidence is increasing worldwide. Although drug-based therapies are progressing, new evidence shows that nutritional approaches play a paramount role in prevention and therapy (1). In this editorial, we emphasize recent evidence and support for a change in paradigm toward integrative dietary strategies in IBD management (Figure 1).

Several researchers have suggested that maternal prenatal diet may be linked to the impact on the offspring's gut health (2). Anneberg et al. analyzed data from 60,274 singleton mother-child dyads recruited in the Danish National Birth Cohort to investigate the relationship between maternal intake of foods and dietary nutrients during pregnancy and the risk of pediatric onset of IBD in offspring, including CD and UC. In this study, frequent maternal intake of organic eggs and dairy products in pregnancy were associated with the potential risks of pediatric-onset CD. No significant relationship was found for UC; however, these findings support a potential role of prenatal diets in long-term gut health in children. Based on this concept, dietary patterns in early adulthood have also been studied in relation to IBD risk. Recent studies have suggested that high consumption of red meat, particularly pork, beef, and lamb, may exacerbate colonic inflammation by altering gut microbiome structure and triggering pro-inflammatory immune reactions (3).

Zhang et al. relapse risk of UC. In their study, the researchers found that a high consumption of red meat, approximately 100 grams per day, was associated with a 65% increased risk of UC development. Based on very low-certainty evidence, high red meat intake may be associated with an increased risk of UC. Nevertheless, the evidence is insufficient to attribute a definite association between the intake of red or processed meat and relapse in UC; the sparse data suggest that processed meat may not increase UC risk to the same extent as red meat. Since diet interacts directly with the gut, more studies are exploring the protective effects of specific probiotic supplements in altering the gut microbiota for improved health outcomes. Probiotics have been effective in the past in reducing antibiotic-associated diarrhea and infectious diarrhea (4).

Wang, Zhou et al. assessed the effect of a complex probiotic product on post-weaning diarrhea in weanling piglets. In the present trial, piglets supplemented with selected strains of probiotics in their diet had significantly less diarrhea. These beneficial effects were primarily due to the enhanced barrier, robust defense system, and lower pathogen load, along with an enriched population of friendly microbes. It is worth noting that the probiotic product, which includes Bacillus subtilis and Bacillus licheniformis, may benefit intestinal health and provide a potent, antibiotic-free method for controlling diarrhea in piglets. Probiotics for IBD-related diarrhea also appear promising, although strain-specific effects and patient variation should be considered. Likewise, the role of micronutrients, including iron, has also been studied for their 2-fold function in meeting physiological requirements as well as potentially modulating intestinal inflammation. Iron replacement is necessary for the treatment of anemia in patients with IBD. However, its effect can vary slightly depending on the form and dose. Redundant iron may exacerbate intestinal injury and inflammation, whereas certain formulations can maintain the efficacy of probiotics and enhance intestinal barrier integrity (5). In a study by Wang, Yang et al., the small intestine is also involved in experimental colitis, and its iron content contributes to the Dextran Sulfate Sodium (DSS)-induced damage/regeneration of the intestines. These results draw attention to the necessity for more careful thought regarding iron supplementation protocols in clinical settings taking into account several factors. In summary, these observations underscore the necessity for individualized iron supplementation strategies that take into account nutritional needs in light of the potential risk of exacerbating gut inflammation. In addition to these micronutrients, integrative therapies that involve pharmacotherapy and nutrition support have also been investigated. Huang et al. assessed the impact of PEN as adjunct therapy to infliximab in CD patients. In the 54-week study, 176 patients were tracked, and PEN was administered as an inducement and maintenance in 77 of them. The findings showed that when added to infliximab treatment, PEN is able to significantly enhance short-term clinical response and sustained endoscopic remission. Such an integrative approach could work by minimizing drug efficacy loss and mucosal healing, offering a strong rationale for the synergistic application of pharmacological and nutritional treatments in CD. Consistently, Traditional Chinese Medicine (TCM) is being applied in drug discovery and disease treatment due to its wide range of biological activities, low toxicity, and few side effects. A number of prior studies have identified therapeutic effects for certain TCM formulas in IBD. Among these, honeysuckle (*Lonicera japonica*) has been developed as a well-known botanical for IBD therapy. Its bioactive components, including chlorogenic acid and luteolin, achieve anti-inflammatory, antioxidant, and microbiota-modulating effects. In addition, exosomes derived from honeysuckle have been shown to repair the intestinal barrier and regulate gut microbiota structure. In a recent article, Muro et al. performed a review on the metabolites from L. japonica as IBD drugs. The underlying mechanisms are summarized, and the contribution of honeysuckle's bioactive ingredients in the prevention and treatment of IBD is emphasized. While not specific to IBD, these mechanisms have been studied in the functional gastrointestinal disorder of IBS. Wang, Zhang et al. assessed the levels of major indole metabolites and the expression of CYP1A1 and Zo-1 in healthy individuals vs. those with IBS-D. The study suggested that colonic mucosa tissues in IBS-D were involved in relatively early events of low-grade inflammation, abnormalities of intestinal barrier function, and visceral sensation. These alterations might be associated with the decreased production of tryptophan-derived indole metabolites, enhanced function of enteric glial cells (EGCs), and inhibition of the aryl hydrocarbon receptor (AHR)/CYP1A1 axis. Special attention should be focused on tryptophan metabolism and the manipulation of AHR and regulatory T cells as potential options for novel IBS treatment strategies. In this setting, nutritional interventions, such as dietary exclusions, specific supplementation, and microbiota-directed therapies, are emerging and changing the therapeutic scenario. The combination of these approaches with traditional therapies may provide the framework for a personalized, efficient, and durable management of IBS. To summarize the evidence mentioned in this Editorial more clearly, we prepared a Table of evidence that includes the main nutritional interventions studied in relation to IBD (Table 1). In this systematic synthesis, and accompanying graphical abstract, we present the evidence for, against, or supporting an effect of dietary factors on the onset, progression, or treatment of IBD to provide a more auditable evaluation that highlights the heterogeneity and quality differences between studies found.

**Table 1:** Evidence table.

**Study (author/s)**	**Design**	**Population/ model**	**Exposure/ intervention**	**Comparator**	**Main findings**	**Limitations**	**Approximate evidence level**
Anneberg et al.	Population-based Danish National Birth cohort.	60,274 mother-child pairs (singleton).	Frequent maternal intake of organic eggs and dairy during pregnancy.	Mothers with organic food consumption during pregnancy and other dietary patterns.	Maternal consumption of organic eggs and dairy is associated with reduced pediatric-onset CD; no significant association with UC.	Observational, residual confounding; diet measured by questionnaires; causality limited.	Low-Moderate.
Zhang et al.	Systematic review.	Human studies on red/processed meat and UC.	High red meat intake (>100 grams/day).	Lower intake.	100 g/day red meat intake linked to ≈65% higher UC risk; very low certainty; processed meat not clearly linked to relapse.	Low quality studies, recall bias, heterogeneity; not causal.	Very Low.
Wang, Zhou et al.	Experimental.	Weaned piglets.	Dietary probiotic supplementation (*Bacillus subtilis, Bacillus licheniformis*).	No probiotics/control diet.	Reduced post-weaning diarrhea; improved barrier, immune response, reduced pathogens, enriched beneficial microbes.	Animal model; strain/dose dependent; limited transferability to IBD humans.	Preclinical.
Wang, Yang et al.	Experimental.	Rodent colitis model (DSS-induced).	Iron supplementation/iron content variation.	Different levels/formulations of iron.	Iron content modulated intestinal damage/regeneration; excess iron worsened injury, and some formulations were beneficial.	Animal model; limited direct translation to clinical IBD; dose/formulation dependent.	Preclinical.
Huang et al.	Clinical longitudinal.	176 CD patients (77 with PEN + infliximab).	Infliximab + PEN	Infliximab alone	Addition of PEN improved short-term response and sustained remission; potential to maintain drug efficacy.	Unclear if randomized; possible selection bias; adherence and PEN details not specified.	Moderate.
Muro et al.	Review of TCM bioactives.	Preclinical and biochemical studies.	Lonicera japonica metabolites (chlorogenic acid, luteolin, exosomes).	—	TCM bioactives show anti-inflammatory, antioxidant, microbiota-modulating effects; barrier repair potential.	Mainly preclinical; lack of clinical trials; variable formulations.	Low-Moderate.
Wang, Zhang et al.	Clinical comparative.	IBS-D patients vs. healthy subjects.	Tryptophan-derived indole metabolites; CYP1A1, Zo-1 expression.	Healthy subjects	IBS-D patients had low-grade inflammation, impaired barrier, reduced indoles, altered AHR/CYP1A1; possible therapeutic target	Correlational; partial translational relevance.	Low-Moderate.

IBD, Inflammatory Bowel Disease; CD, Crohn's disease; UC, ulcerative colitis; DSS, Dextran Sulfate Sodium; PEN, partial enteral nutrition; TCM, Traditional Chinese Medicine; IBS-D, Diarrhea-predominant irritable bowel syndrome; Zo-1, Zonula Occludens-1; AHR, aryl hydrocarbon receptor.
